# Changes in C57BL6 Mouse Hippocampal Transcriptome Induced by Hypergravity Mimic Acute Corticosterone-Induced Stress

**DOI:** 10.3389/fnmol.2016.00153

**Published:** 2016-12-26

**Authors:** Alice Pulga, Yves Porte, Jean-Luc Morel

**Affiliations:** ^1^Université de Bordeaux, Institut des Maladies Neurodégénératives, UMR 5293Bordeaux, France; ^2^Centre National de la Recherche Scientifique, Institut des Maladies Neurodégénératives, UMR 5293Bordeaux, France

**Keywords:** aging, chronic stress, acute stress, gravity, hippocampus, transcriptome, Illumina

## Abstract

Centrifugation is a widely used procedure to study the impact of altered gravity on Earth, as observed during spaceflights, allowing us to understand how a long-term physical constraint can condition the mammalian physiology. It is known that mice, placed in classical cages and maintained during 21 days in a centrifuge at 3G gravity level, undergo physiological adaptations due to hypergravity, and/or stress. Indeed, an increase of corticosterone levels has been previously measured in the plasma of 3G-exposed mice. Corticosterone is known to modify neuronal activity during memory processes. Although learning and memory performances cannot be assessed during the centrifugation, literature largely described a large panel of proteins (channels, second messengers, transcription factors, structural proteins) which expressions are modified during memory processing. Thus, we used the Illumina technology to compare the whole hippocampal transcriptome of three groups of C57Bl6/J mice, in order to gain insights into the effects of hypergravity on cerebral functions. Namely, a group of 21 days 3G-centrifuged mice was compared to (1) a group subjected to an acute corticosterone injection, (2) a group receiving a transdermal chronic administration of corticosterone during 21 days, and (3) aged mice because aging could be characterized by a decrease of hippocampus functions and memory impairment. Our results suggest that hypergravity stress induced by corticosterone administration and aging modulate the expression of genes in the hippocampus. However, the modulations of the transcriptome observed in these conditions are not identical. Hypergravity affects *per-se* the hippocampus transcriptome and probably modifies its activity. Hypergravity induced changes in hippocampal transcriptome were more similar to acute injection than chronic diffusion of corticosterone or aging.

## Introduction

Stress is known to modulate memory by affecting the hippocampus functions. As reviewed recently (Kim et al., 2015; Pearson-Leary et al., 2015), norepinephrine and glucocorticoids act on memory via modulations of neuronal functions, neurogenesis, and glial cells. The effects of glucocorticoids depend on their concentration, synthesis, and turn-over. Moreover, as suggested by the high density of glucocorticoids receptors, the hippocampus, together with the amygdala, represent one of the main targets of glucocorticoids (Reul and De Kloet, 1985). Indeed, infusions of corticosterone (or its analogs) in the hippocampus affect memory performance in different paradigms (Micheau et al., 1984; Roozendaal and McGaugh, 1997). Glucocorticoids appear to be necessary for memory consolidation, yet while acute post-training injection of low doses enhance performance in aversive and spatial tasks, higher doses or chronic treatment with low doses impair memory and hippocampal functions (McGaugh and Roozendaal, 2002; Marks et al., 2015). However, a very high dose of corticosterone is able to mimic the memory impairment observed in the post-traumatic stress disorder (PTSD; Kaouane et al., 2012), which is characterized by hypermnesia for the core traumatic event associated with a memory deficit for peritraumatic contextual cues.

Besides the typical events leading to stress, an increase of corticosterone levels in plasma samples has been observed in rats (Petrak et al., 2008) and mice (Gueguinou et al., 2012) exposed to conditions of altered gravity.

Life is conditioned by the gravity vector, from conception to adult stages. Adult mammals, during their whole life, need to integrate this force to coordinate the communication between organs and maintain their physiology in a balanced steady-state. The alteration of gravity is a model used to shed light on fundamental processes implicated in environmental adaptation influencing the genomic expression, and it may be helpful to understand how organisms can evolve and adapt to their life on Earth. However, during space flights, astronauts undergo gravity modifications. The most remarkable effects induced by microgravity observed after spaceflights in both humans and rodents are bone decalcification, decrease of musculature, and cardiovascular deconditioning, but they also show spatial disorientation, depressive-like, and cognitive disorders (Porte and Morel, 2012). In space, astronauts as animal models are exposed to several healthy risks, such as secondary radiations (due to high energy protons from solar radiation), modification of light-dark cycle, confinement, and modification of gravity level. In order to isolate the impact of gravity and confinement from the radiation exposures, we used a model consisting of the confinement of mice in a centrifuge, which creates a modification in gravity level.

Using the same protocol, a behavior analysis using a Morris water maze 15 days after exposure to hypergravity (21 days, 3G) suggested that the memory processes could be affected (Bojados and Jamon, 2014). In the same conditions, corticosterone blood levels also revealed that the stress level in these mice was increased in the hours following centrifugation (Gueguinou et al., 2012). The increase of corticosterone levels observed in this study could be ascribed to (1) a long lasting stress induced by the hypergravity and/or (2) an acute stress due to the centrifuge brake.

Since gravity changes induce modifications of genome expression in several tissues (Morel et al., 2014), we hypothesized that hypergravity as well as stress (induced by acute or chronic administration of corticosterone) could affect the genome expression in the hippocampus, with putative effects on memory. In fact, modifications of spatial memory have been previously suggested in rat exposed to hypergravity (Mitani et al., 2004). To assess the relationship between centrifugation and stress effects on hippocampal structure, we performed a transcriptomic analysis of the hippocampus of mice which underwent either centrifugation or chronic and acute corticosterone administration.

Finally, since deleterious effects induced by gravity modifications have been presented as an acceleration of aging (Vernikos and Schneider, 2010), we compared the hypergravity situation with aging effects on the hippocampus transcriptome (Vernikos and Schneider, 2010); moreover, aging is also associated with an increase of plasma corticosterone levels and modifications in hippocampus-dependent processes (Lo et al., 2000; Garrido et al., 2012a,b).

## Materials and methods

### Animals, centrifugation, and corticosterone treatments

Eight week-old C57BL/6J male mice were purchased from Charles River (Les Oncins, 69,210 Saint Germain sur l'Arbesle, France) and housed in standard cages (four mice per cages, 36 × 20 × 14 cm) under standard conditions (22°C, 55% humidity, 12/12 h light-dark cycle) with free access to standard food and water. Mice were habituated to animal room for 2 weeks prior to beginning testing.

Mice were divided into different groups described as follows: 24 mice (six cages) were placed in the centrifuge at 3G for 21 days (3G group = hypergravity); 24 mice were placed in normogravity (1G group = confinement) in the centrifuge room and confined in the same gondola as used in the centrifuge. The cages were supplied with enough food and water to allow an uninterrupted 21 days centrifugation/confinement period. This part of the experiment was performed in the animal facility containing the centrifuge (Hôpital de la Timone; Marseille, France; Marc Jamon was responsible for centrifugation).

A group of 10 mice were implanted subcutaneously with Matrix-Driven Delivery (MDD) pellets (Innovative Research of America, Sarasota, FL, USA) containing 10 mg of corticosterone, allowing the constant and continuous delivery of corticosterone during 21 days (CC group). Likewise, a control group of 10 mice were implanted with the placebo pellets during 21 days (PL group), in order to rule out a putative confounding effect of implantation and surgery in the CC group. One mouse treated with placebo died before the end of the protocol. Furthermore, 10 mice (13 weeks old, aged-matched with CC and PL groups on the day of euthanasia) received a single intraperitoneal injection of corticosterone (in 2-hydroxypropyl-β-cyclodextrin complex; 1.5 mg/Kg; in a volume of 0.1 ml/10 g bodyweight; group AC), 1 h before euthanasia as described previously (Kaouane et al., 2012).

Finally, a group of 15 mice of 22 month (AGE group) was also included in the study to compare the effect of aging to hypergravity and corticosterone-induced stress.

CC, PL, AC, and AGE mice were housed in the animal facility of our laboratory, in Bordeaux, France.

All animal groups are summarized in Table 1.

**Table 1:** **Design of experimental groups**.

**Group**	**3G**	**1G**	**AC**	**CC**	**PL**	**AGE**
*n*	24	24	10	10	10	15
Age		10 weeks at the beginning of the protocol				80 weeks
Gravity level	3 × G	Normogravity (1G)				
Treatment	Centrifuge	None	Corticosterone Intraperitoneal	Corticosterone	Placebo	None
				Subcutaneous 21 days		

The project has been validated by the French Ministry of Research in accordance with the European Community and French guiding principles (C2EA-50 (Bordeaux) C2EA-14 (Marseille)). The principal investigator is authorized by French authorities to perform animal experiments (n° C33-01-029).

Mice were euthanized in the same time window (9–11 a.m.) by a lethal dose of pentobarbital, brains were extracted from the skull and blood was collected. Hippocampi were dissected and placed in 2 mL tubes containing 1 mL of tri-reagent (TR-118, MRC, Cincinatti, OH) and 10–12 ceramic bead (SiLibeads, ZS, 2–2.2 mm diameter, Labomat Essor, Saint-Denis France) and frozen in dry ice. Samples of group 1G and 3G were transported to Bordeaux in dry ice. All samples were prepared for transcriptomic analysis as described below.

### Preparation of RNA samples for transcriptomic assays

After disruption and homogenization of all samples with minilys (Precellys, distributed by Ozyme France, Montigny le Bretonneux) in tri-reagent (Molecular Research Center, Inc., Cincinnati, USA), isolation of the total RNA from each hippocampus was performed following the supplier procedures. RNA integrity and purity were verified by using RNA HighSens Analysis Kit (Experion, BioRad, Marne-la-Coquette, France) and the concentration of RNA was measured with spectrophotometry (NanoDrop Technologies, Wilmington, DE) for each hippocampus. We determined that the total RNA in one hippocampus was not sufficient to be sequenced by Illumina. Thus, the RNAs from three to eight mice were pooled in order to generate three samples for each experimental condition (Supplementary Figure 1). Samples were sent to GATC, the company which verified the quality of the samples and performed the transcriptomic analysis using Genome Sequencer Illumina HiSeq2000^*^ (sequence mode single read 1 × 50 bp), and sequences were mapped. The quantities of sequences obtained in each sample were sufficient to perform a statistical analysis (Supplementary Figure 2).

### Quantitative real-time polymerase chain reaction after transcription (RT-qPCR)

Reverse transcription (RT) reaction was carried out using the iScript advanced kit (Bio-Rad). Obtained cDNA were amplified using the primers designed with Quantprime software (Arvidsson et al., 2008). The sequences of primers finally used are indicated as forward primer/reverse primer [accession number], current name of the gene. CATTACCCAGGTATTGCTGTTCCC/TAGAGCTCCCAGTGTGCTGTAG [NM_001033260.1], stox1; CGTTCCATGAATTCGCGGATGTG/TGCTGTAGGAGTATGGGCTGAG [NM_013697.4], ttr; TTCCCAGTGGTGACTGTCCAAG/TCTTCGCCACAAAGGCACCTG [NM_001110227.1], kcnj13; TCTCTGGTGATCAGGATACAGGTG/AGCCGTGGAGAAGATCTGAGAC [NM_022310.2], hspa5; AGAATCCAGGGTGCAGGTATGG/TCTTGGCAGGCCTCACTTTGTTC [NM_144841.3], otx2; TTCACTCTGGGAAATATGCACAGG/GTGGCCACTTGCACATTGTAG [NM_008491.1], lcn2; TGGCCTGAATCACTTGGACAGC/ATCATATTGCCCAGGAGCCTGAAG [NM_008220.4], hbb-b1. The real time qPCR experiments were performed with the SsoADV Univer SYBR Green Supermix (Bio-Rad) in the CFX96 thermocycler (Bio-Rad). The specificity of the amplification products was confirmed by melting curve analysis. All samples were analyzed in duplicates. PCR efficiency was calculated from the slope of the standard curve. Gene expression levels were calculated using the 2-dCt method normalized by reference gene (stox1) that is steadily expressed in transcriptomic analysis and we have verified also by RT-qPCR.

### Corticosterone assays

Blood was drawn just after the cervical dislocation. After 10 min, samples were centrifuged during 15 min at 1000 g and supernatants were collected and stored at −20°C before assays. The quantification of endogenic mouse corticosterone in blood samples was performed using EIA kit DetectX Corticosterone (Arbor Assays, Michigan distributed by Euromedex) following the instructions of the supplier: samples and standards solutions were diluted (1:100) in the buffer and placed in the plate in duplicates. The reaction buffer containing anti-corticosterone antibody was added and after 2 h of incubation the chemoluminescent subtract was added. The chemoluminescence was measured with the Optima apparatus (BMG Labtech, Champigny sur Marne, France).

### Statistical analysis

The first steps of transcriptomic data analysis were performed by GATC. To validate the statistical analysis, *p*-values were corrected using the Benjamini and Hochberg procedure controlling false discovery rate (FDR). Supplementary statistical analyses were performed with Graphpad prism software (Graphpad software Inc., La Jolla, CA). Data were expressed as means ± S.E.M.; *n* represents the number of tested animals. The samples were compared pair by pair with *t*-test and with one-way ANOVA. The *P* < 0.05 were considered as significant and indicated by ⋆ in figures.

### Database queries

The list of proteins was analyzed using Database for Annotation, Visualization, and Integrated Discovery (DAVID; https://david.ncifcrf.gov), in order to identify the associated pathways and function of the different genes modified in the experimental conditions. The query was made as proposed: *sp-pir-keywords* for functional categories and *biocarta, KEGG pathway*, and *panther pathway* for signaling pathways. Finally, MGI database was used to suggest the putative phenotype outcome of changes induced by hypergravity.

## Results

### Corticosterone levels

**Figure 1:**
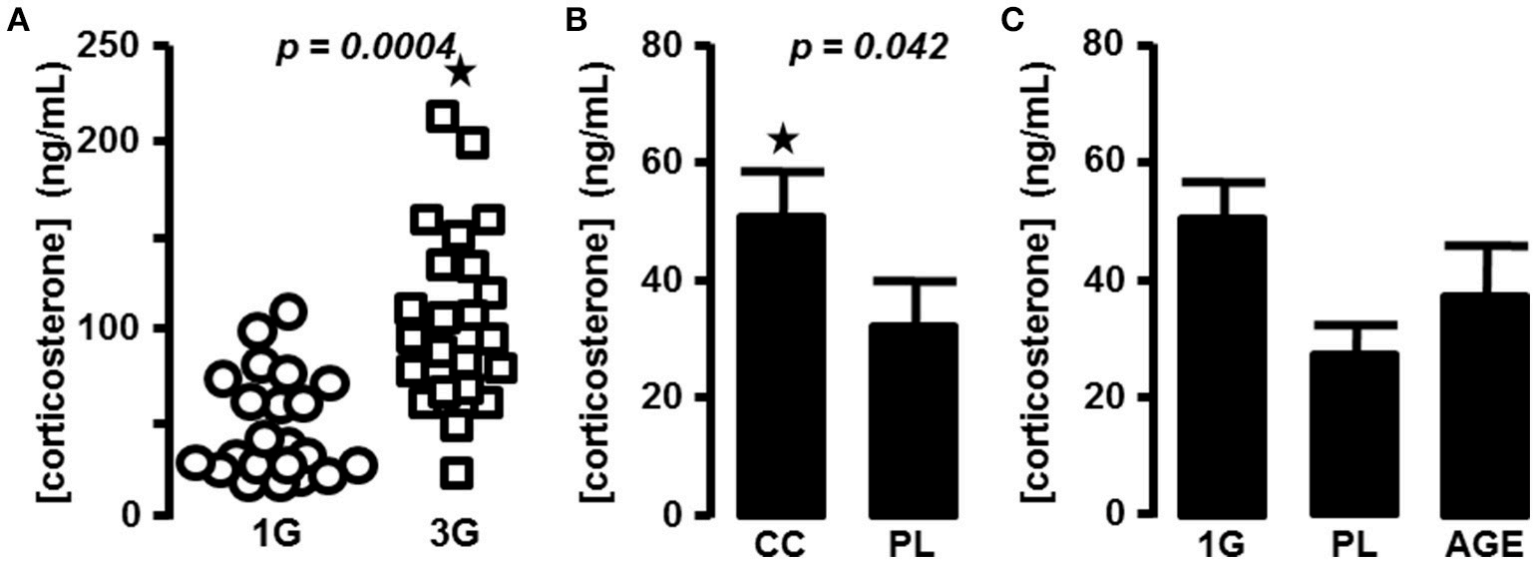
**Blood corticosterone concentrations. (A)** in 1G- (circle) and 3G- (square) exposed animals; each dot represents the mean of the duplicate measured for each animal. **(B)** Mean of corticosterone concentration measured in placebo and chronic corticosterone treatment. The results were statistically compared with *t*-test; ⋆*p* < 0.05. **(C)** Mean of corticosterone concentration measured in placebo, 1G and aged mice groups.

As described previously in mice centrifuged in the same device (Gueguinou et al., 2012), we measured an increase of the plasma level of corticosterone during the 2 h following a period of 21 days in the centrifuge at 3G (59.4 ± 8 ng/mL vs. 113 ± 11.6 ng/mL for 1G and 3G groups, respectively; *n* = 24 for each group; *p* = 0.0004). We also noticed that in the 3G group the variability of individual measurements was increased (Figure 1A). We therefore decided to complete the statistical analysis by the modified Thompson tau test to determine the outliers' points. The result remained unchanged (52.5 ± 7 ng/mL, *n* = 22 vs. 107 ± 10 ng/mL, *n* = 23 for 1G and 3G groups, respectively; *p* = 0.0001).

To compare the effects of hypergravity on the transcriptome to those observed in stress conditions, we treated mice chronically with corticosterone pellets. The levels of corticosterone in blood of animals treated with chronic corticosterone and placebo were evaluated (33.6 ± 7 ng/mL, *n* = 9 vs. 51.3 ± 8.2 ng/mL, *n* = 10 for PL and CC groups, respectively; *p* = 0.118), and the *p*-value was ameliorated by the use of the modified Thompson tau test (28.4 ± 5.3 ng/mL, *n* = 8 vs. 51.3 ± 8.2 ng/mL, *n* = 10 for PL and CC groups, respectively; *p* = 0.042), as reported in Figure 1B.

The acute intraperitoneal injection of corticosterone induced an increase of blood corticosterone concentration close to 10-fold more than in the 3G group (1201.8 ± 232.7 ng/mL, *n* = 10; one-way ANOVA, *p* = 0.001 in comparison with all other groups).

Finally, in aged mice corticosterone levels were not significantly affected in comparison with the placebo group used as control younger mice (33.6 ± 7 ng/mL, *n* = 9 vs. 52.7 ± 17.1 ng/mL, *n* = 15; *p* = 0.193. After the modified Thompson tau test, values were 28.4 ± 5.3 ng/mL, *n* = 8 vs. 38.0 ± 9.3 ng/mL, *n* = 14; *p* = 0.383; for PL and AGE groups, respectively, Figure 1C).

### Transcriptomic analysis

The Illumina analyses revealed the presence of 33,842 different sequences in the transcriptome of the hippocampus. The statistical comparison showed that the expressions of 82 transcripts were affected by hypergravity compared to normogravity, corresponding to 77 identified genes, while 5 sequences were not associated to known genes. In the other conditions, some genes were associated with several sequences; we have mentioned them in Table 2 as repeated sequences. After excluding sequences not associated with gene names and repeated sequences, the expressions of 110 transcripts were modified by acute injection of corticosterone compared to placebo, 102 by chronic administration of corticosterone compared to placebo, and finally 3308 by aging compared to young mice placed in normogravity (Table 2).

**Table 2:** **Number of transcripts affected by experimental conditions**.

**Group comparison**	**3G vs. 1G**	**AC vs. PL**	**CC vs. PL**	**1G vs. AGE**
Sequences	33,842	33,842	33,842	33,842
Fail	1061	994	943	892
Lowdata	93	93	93	93
No test	4049	1122	1891	1959
Seq analyzed	28,639	31,633	30,915	30,898
P < 0.05	82	131	108	4095
Not associated with genes	5	20	6	592
Repeated sequences	0	1	0	195
Identified sequences	77	110	102	3308

**Figure 2:**
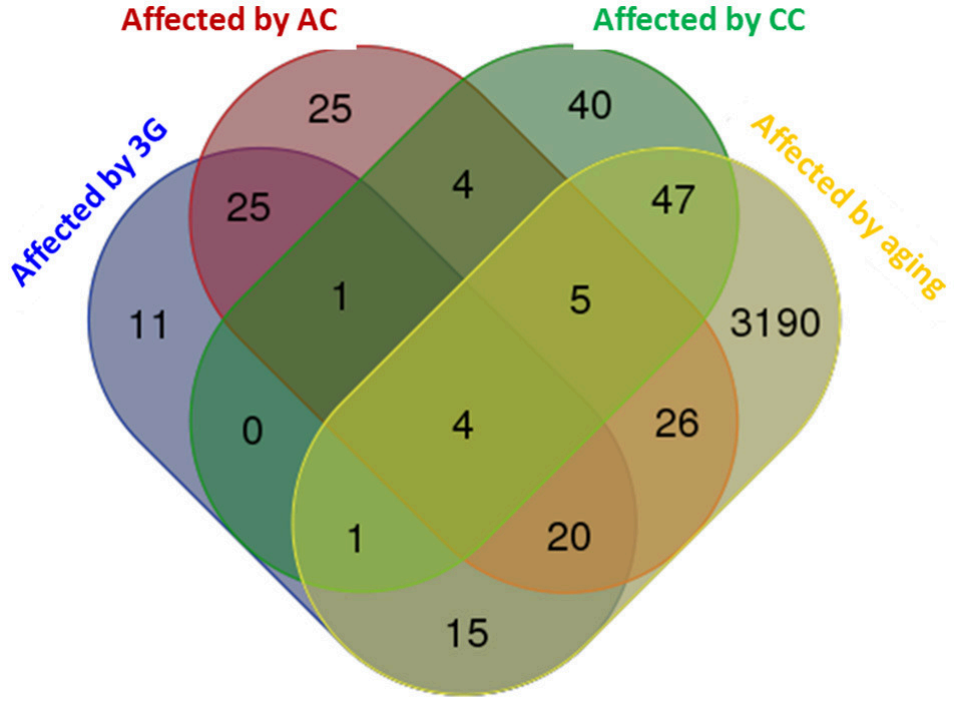
**Venn diagram comparing the effects of all experimental conditions on hippocampus transcriptome**. The diagram was produced by the software available on http://bioinformatics.psb.ugent.be using the lists of genes statistically affected in each experimental condition. The number of genes affected by the experimental conditions is indicated in each colored subset.

The global modifications of transcriptome produced by the different experimental conditions were compared and summarized in a Venn diagram in Figure 2. The profile of the hypergravity effect was qualitatively and quantitatively more similar to the effect of the acute injection of corticosterone than to the long lasting delivery of corticosterone.

**Figure 3:**
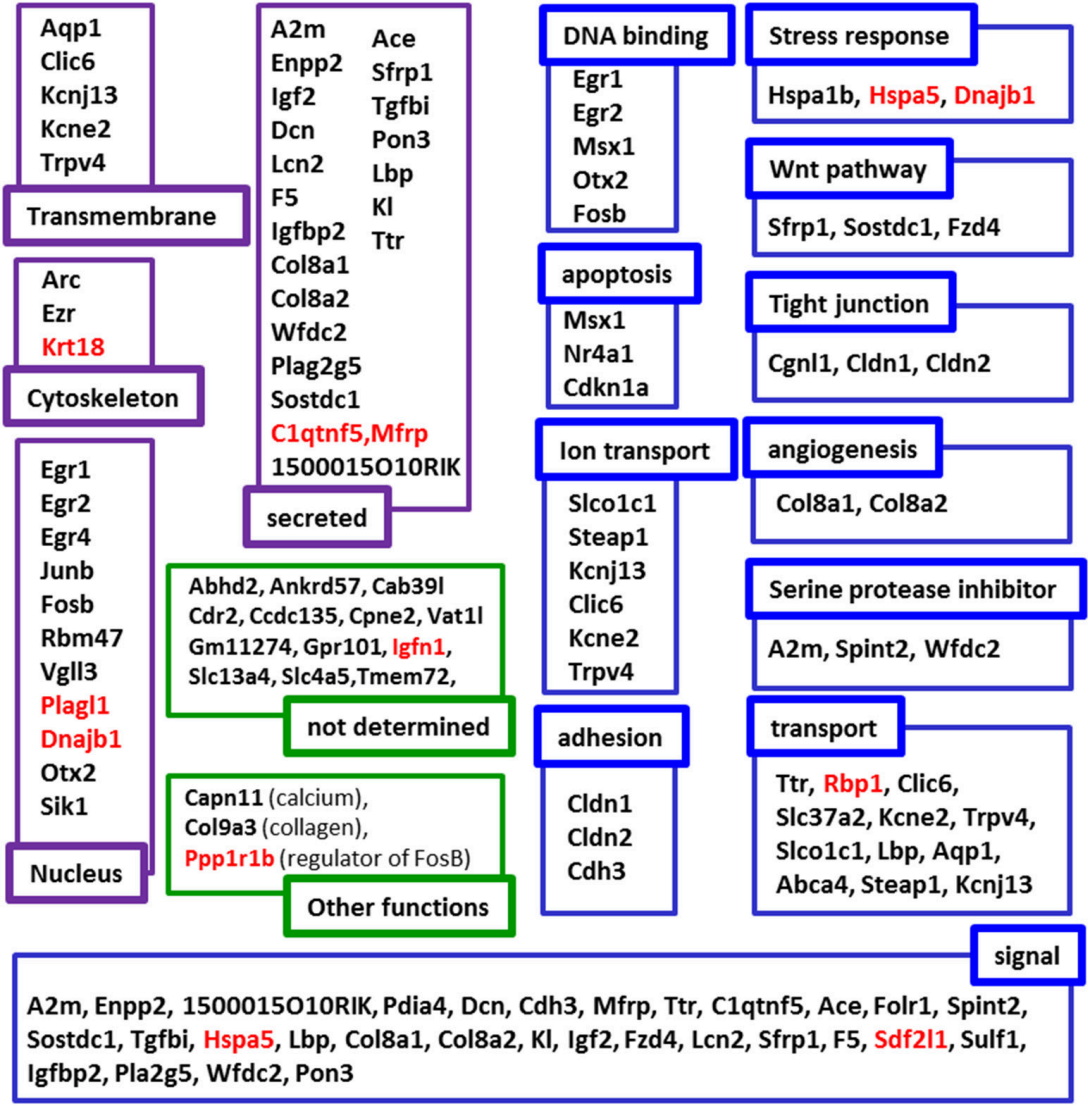
**Putative functions and localizations of proteins encoded by genes affected by 3G**. The list of genes modified by hypergravity was analyzed using DAVID database and genes listed in DAVID were further grouped according to the localization (purple subsets) and the functions (blue subsets) of their encoding proteins. In green, genes not identified by DAVID were grouped in “not determined” subsets; genes identified in other lists were indicated in “other function subsets” with the function indicated in parenthesis. Genes' names in red indicate those only affected by 3G.

To map the putative effects of hypergravity, we used the DAVID database. Seventy six genes were analyzed and dispatched in 37 groups. Figure 3 summarized how the genes could be grouped according to their known functions and cellular localization. The detailed DAVID queries are described in Supplementary Table 1.

The statistical comparisons between control and experimental groups were grouped in Supplementary Table 2. In details, the increases of expression levels of eight transcripts and the decreases of the expression of two transcripts were specifically affected by hypergravity only (Table 3). The Table 4 summarized the comparison between the modification of transcriptome induced by hypergravity and the other conditions (AC, CC, and AGE). Hypergravity and acute injection of corticosterone similarly affected the expression of 49 transcripts (but for 26 of them, a marked difference is noticeable between PL and 1G groups), and three transcripts were altered in opposite ways when compared respectively to normogravity and PL controls (Table 5).

**Table 3:** **Transcript expression levels affected only by 3G compared to normogravity (1G) (decreased in italic; all others increased)**.

**Gene**	**Locus**	**Group 1G**	**Group 3G**	**log2(fold_change)**	***p*****-value**	***q-*****value**
*Plagl1*	*10:12810656-12851500*	*6.098*	*4.172*	*−0.5474*	*1.274E-04*	*0.04617*
Igfn1	1:137878484-137880871	0.241	0.961	1.9973	1.137E-04	0.04340
Hspa5	2:34627514-34655580	45.661	61.084	0.4198	3.614E-07	0.00025
Dnajb1	8:86132079-86135915	19.787	26.955	0.4460	1.794E-06	0.00104
Sfrp1	8:24522292-24560100	0.993	1.637	0.7208	9.436E-05	0.03652
C1qtnf5, Mfrp	9:43909713-43917327	5.721	11.043	0.9488	1.253E-06	0.00081
Rbp1	9:98325199-98347038	12.090	18.123	0.5840	4.914E-05	0.02100
Ppp1r1b	11:98209550-98219164	15.030	19.941	0.4079	5.064E-05	0.02132
Krt18	15:101861304-101862451	0.507	3.744	2.8841	6.800E-05	0.02704
Sdf2l1	16:17130229-17132410	10.438	17.327	0.7311	1.432E-07	0.00011

*The values are expressed as Fragments Per Kilobasepair per Million (FPKM) of the gene in sample with respective p-value and q-value, which is the FDR-adjusted p-value*.

**Table 4:** **Effect of hypergravity on gene expression**.

**Gene**	**Group 3G- log2(fold-change)**	**Group AC- log2(fold-change)**	**Group CC- log2(fold-change)**	**Group AGE- log2(fold-change)**
Sulf1	1.30335	2.54783		−0.826247
F5	1.93139	3.95502		2.32089
1500015O10Rik	1.75095	2.99507		0.834877
Igfbp2	0.880923	1.56046		0.600096
Kcnj13	1.97071	4.47569		3.10135
Ankrd57	0.594451	0.867332		
Egr2	1.21264			3.06703
Dcn	−0.573591			2.21481
Ace	1.41923	2.55624	0.632951	
Sostdc1	1.40975	2.78276		0.981031
Gm11274	−0.974941			2.23011
Tgfbi	0.866265	1.06921		
Otx2	1.49754	3.16522		−1.31388
Cab39l	0.466337	0.803683		
Nr4a1	0.475274			1.73113
Enpp2	1.4905	2.51697		
Arc	0.634743			2.49228
Cldn1	1.38973	1.75401		
Col8a1	1.5564	3.57162		
Vgll3	−1.19884	2.09066	1.56852	1.75075
Kcne2	2.07954	4.69944		2.9754
Clic6	1.68149	3.52024		
Cdkn1a	0.733637	0.939498		
Sik1	0.557851	1.03223		−0.519891
Hspa1b	0.849894		0.567793	1.65455
Capn11	−1.65456			3.03854
Ezr	0.388112			0.974911
Ttr	2.18462	4.38164	0.964938	2.88912
Egr1	0.566316			1.7932
Lbp	1.37942	1.97479		
Wfdc2	2.03256	3.83212		
Col9a3	1.23352	1.64091		
Lcn2	1.72437	−2.70037	1.6198	2.3442
Abca4	1.51953	2.96413		
Col8a2	1.24666	2.33567		
Pla2g5	1.75196	2.39452		
Trpv4	1.7617	2.94136		
Kl	1.23118	2.05559		3.02789
Msx1	0.940395			1.4076
Steap1	1.83045	3.40711		
Rbm47	1.65002	2.80976		
Tmem72	1.8963	5.47826		
A2m	0.701867	1.41704		−1.87054
Slco1c1	0.355327			0.628088
Slc13a4	0.764586	1.97186		1.66465
Pdia4	0.462279			0.574577
Pon3	0.938093	1.95674		
Aqp1	2.3098	4.09336		2.03643
Slc4a5	2.30275	4.71954		1.95522
Egr4	0.538811			2.43387
Folr1	1.99678	3.64892		1.95393
Cdr2	0.604981	1.10548		−1.43491
Igf2	0.706057	1.52015		1.61939
Fosb	0.818794			1.39926
Spint2	0.626409	1.05814		
Abhd2	0.319449	0.599141		0.509787
Fzd4	0.542651	0.784285		
Cdh3	2.78157	4.28946		
Vat1l	0.649073	1.36456		−2.60184
Junb	0.471423			2.56019
Cpne2	−0.38987	0.661188	0.378233	0.472237
Ccdc135	1.10931	2.51124		
Slc37a2	0.917782	1.25844		
Cgnl1	0.752135	1.08226		
Cldn2	2.13111	4.68814		2.30732
Gpr101	−0.598134			−0.677419

*In red box increase of expression, in green box decrease of expression and the gray box indicated that the gene expression is not modified. The values log2(fold-change) between 3G and 1G (Group 3G); AC and 1G (Group AC); CC and 1G (Group CC); AGE and 1G (Group AGE)*.

**Table 5:** **Transcript expression levels affected by both 3G and AC compared to 1G and placebo, respectively**.

**Gene**	**Locus**	**Group 1G**	**Group 3G**	**Group PL**	**Group AC**
Sulf1	1:12682400-12851259	1.98	4.888	1.024	5.989
Igfbp2	1:72871044-72899041	15.406	28.372	9.198	27.13
Col9a3	2:180332941-180356965	5.792	13.619	3.828	11.937
Lbp	2:158132322-158158118	2.452	6.38	1.687	6.633
Pla2g5	4:138355177-138375157	0.345	1.163	0.202	1.064
Kl	5:151755320-151796908	5.741	13.477	3.428	14.25
A2m	6:121586106-121629223	0.896	1.457	0.697	1.862
Pon3	6:5167314-5206224	0.808	1.549	0.49	1.901
Cdr2	7:128100553-128125695	3.29	5.005	2.772	5.964
Igf2	7:149836670-149846925	18.885	30.807	10.796	30.966
Spint2	7:30041283-30067035	7.703	11.891	5.302	11.039
Abhd2	7:86418109-86510391	13.81	17.232	12.624	19.122
Fzd4	7:96552850-96561625	1.873	2.729	1.836	3.162
Vat1l	8:116729446-116897994	6.811	10.681	5.001	12.877
Slc37a2	9:37035195-37063010	0.624	1.178	0.495	1.183
Cgnl1	9:71474312-71619366	2.871	4.835	2.367	5.012
Ankrd57	10:58684611-58689177	2.013	3.04	1.622	2.959
Tgfbi	13:56710929-56740717	1.199	2.185	1.124	2.359
Cab39l	14:60059798-60167840	7.41	10.238	6.317	11.027
Enpp2	15:54670216-54785330	93.005	261.333	51.59	295.292
Cldn1	16:26356751-26371926	0.692	1.814	0.714	2.409
Cdkn1a	17:29230693-29237671	3.861	6.42	4.491	8.613
Sik1	17:31981268-31992685	2.661	3.917	2.023	4.138
**F5**	**1:166081948-166150560**	**1.042**	**3.977**	**0.3036**	**4.709**
**Kcnj13**	**1:89223550-89347501**	**0.765**	**2.999**	**0.1747**	**3.887**
**1500015O10Rik**	**1:43787414-43799794**	**4.944**	**16.642**	**1.926**	**15.353**
**Wfdc2**	**2:164387945-164393983**	**0.706**	**2.887**	**0.196**	**2.797**
**Abca4**	**3:121746980-121882965**	**1.112**	**3.188**	**0.442**	**3.451**
**Col8a2**	**4:125973864-125991692**	**0.988**	**2.345**	**0.488**	**2.464**
**Trpv4**	**5:115072163-115108406**	**1.419**	**4.812**	**0.533**	**4.097**
**Steap1**	**5:5736328-5749282**	**0.791**	**2.814**	**0.266**	**2.823**
**Rbm47**	**5:66407880-66437455**	**0.263**	**0.826**	**0.128**	**0.9**
**–**	**6:116629116-116635214**	**0.527**	**2.175**	**0.037**	**2.371**
**Tmem72**	**6:116641552-116666943**	**0.338**	**1.257**	**0.074**	**3.298**
**Slc13a4**	**6:35217813-35258151**	**2.567**	**4.361**	**1.262**	**4.949**
**Aqp1**	**6:55286159-55298535**	**0.941**	**4.664**	**0.274**	**4.67**
**Slc4a5**	**6:83187361-83254934**	**0.649**	**3.2**	**0.123**	**3.233**
**Folr1**	**7:108988596-109017870**	**4.438**	**17.713**	**1.327**	**16.641**
**Cdh3**	**8:109079141-109079838**	**0.131**	**0.903**	**0.043**	**0.844**
**Ccdc135**	**8:97579011-97602287**	**2.471**	**5.331**	**1.082**	**6.166**
**Ace**	**11:105829288-105851266**	**3.804**	**10.173**	**1.706**	**10.035**
**Sostdc1**	**12:37040651-37045032**	**2.486**	**6.606**	**0.946**	**6.513**
**Otx2**	**14:49277354-49287141**	**1.418**	**4.005**	**0.483**	**4.332**
**–**	**14:76651174-76652137**	**5.640**	**14.079**	**1.432**	**10.791**
**Col8a1**	**16:57624392-57678659**	**0.553**	**1.626**	**0.181**	**2.149**
**Kcne2**	**16:92292609-92298528**	**1.715**	**7.248**	**0.234**	**6.082**
**Clic6**	**16:92498344-92541580**	**2.629**	**8.434**	**0.831**	**9.539**
**Ttr**	**18:20818884-20832827**	**375.259**	**1705.95**	**69.393**	**1446.51**
**Cldn2**	**X:136335278-136345917**	**0.831**	**3.641**	**0.155**	**4.004**
***Lcn2***	***2:32240155-32243278***	***0.958***	***3.167***	***2.081***	***0.32***
***Cpne2***	***8:97057028-97094435***	***18.178***	***13.873***	***9.387***	***14.844***
***Vgll3***	***16:65828218-65866609***	***1.016***	***0.442***	***0.193***	***0.821***

*In bold: when groups 1G and PL presented values different of more than two-fold, i.e., confinement effect; in italic: when the effects between 1G and 3G and between PL and AC were in opposite ways; for all other sequences, there was no difference between 1G and PL, and both comparisons showed similar increases. The values are expressed as Fragments Per Kilobasepair per Million (FPKM) of the gene in sample*.

The chronic administration of corticosterone and hypergravity affected the expression of four transcripts similarly (but for two of them, a marked difference is noticeable between PL and 1G groups) and showed opposite effects on the expression of two transcripts compared respectively to normogravity and PL controls (details in Supplementary Table 2).

Hypergravity and aging together affected the expression of 45 transcripts, of which 13 were similarly modified and 32 were affected contrariwise compared to adult mice in normogravity (details in Supplementary Table 2). Hypergravity and the acute injection of corticosterone resulted to be the closest experimental conditions in modulating the hippocampal transcriptome. Table 6 lists the 47 transcripts affected by acute injection of corticosterone but not by hypergravity (but for 15 of them, a marked difference is noticeable between PL and 1G groups).

**Table 6:** **Transcript expression levels affected by AC but not by 3G and compared to 1G and placebo, respectively (in bold: when groups 1G and PL presented values different of more than two-fold)**.

**Gene**	**Locus**	**Group PL**	**Group AC**	**Group 1G**	**Group 3G**
Prelp	1:135806851-135817995	6.119	10.092	8.991	11.076
Rgs16	1:155587388-155592662	2.468	8.272	2.594	3.453
Plcb4	2:135485156-135840371	4.181	8.883	4.704	4.926
Bmp7	2:172693519-172765977	1.034	2.264	1.743	2.322
Sox18	2:181404542-181406350	2.634	1.088	3.305	3.348
Sgms2	3:131033242-131153082	0.501	1.128	0.690	1.091
Txnip	3:96361859-96365785	6.449	15.570	6.819	9.094
Rims3	4:120527441-120569406	8.236	13.792	8.760	9.828
Id3	4:135699645-135701474	18.669	10.784	18.856	21.987
Alpl	4:137297663-137352249	1.573	3.168	2.559	3.427
Errfi1	4:150229180-150243052	11.745	18.439	12.633	13.599
Hes5	4:154335009-154336494	3.570	1.047	4.734	3.688
Cit	5:116357249-116459005	6.297	11.750	7.865	9.303
AC113316.1	5:147043206-147114511	18.642	291.688	32.813	34.458
Apold1	6:134933638-134936893	0.947	2.796	1.037	1.597
Mdfic	6:15671340-15752163	0.705	2.036	1.041	1.737
Hbb-b1	7:110975050-110982005	355.809	546.092	462.061	501.292
Syt9	7:114514498-114692169	1.253	2.976	1.742	1.506
Zfp36	7:29161807-29164279	1.273	3.202	1.869	2.431
Plekhf1	7:39005604-39012997	1.458	3.225	1.718	1.885
Tnnt1	7:4456181-4466249	0.337	3.947	0.345	0.443
Slc17a6	7:58877006-58926498	3.873	8.414	3.278	3.980
Lars2	9:123276062-123371876	13.944	115.222	14.733	14.913
Amotl1	9:14346410-14447921	2.982	6.149	3.287	3.750
Slc37a2	9:37035195-37063010	0.495	1.183	0.624	1.178
Zic1	9:91252845-91260745	1.643	5.271	2.526	3.483
D10Bwg1379e	10:18299938-18463830	1.924	0.000	2.127	2.047
Perp	10:18564896-18576876	1.122	2.622	1.666	2.276
Sgk1	10:21601862-21719710	17.961	44.267	18.984	25.136
Ddit4	10:59412417-59414734	22.596	35.464	24.492	27.538
Arl4d	11:101526844-101529145	5.293	10.395	6.036	7.725
Adra1b	11:43588132-43649883	0.695	1.875	0.781	0.952
Ramp3	11:6558299-6577482	1.685	6.132	1.948	2.485
Rpl23a	11:77990411-77997079	9.030	2.244	8.150	8.976
Coch	12:52694347-52706789	1.798	4.422	2.338	3.326
Nfkbia	12:56590395-56593615	8.681	21.278	9.183	11.524
Abhd12b	12:71255075-71285422	0.092	1.096	0.132	0.110
Gadd45g	13:51942029-51943872	11.307	19.046	11.666	14.819
Slitrk6	14:111147307-111154449	0.114	0.589	0.116	0.157
Slc39a4	15:76446259-76447453	0.238	1.038	0.439	0.884
Apol7e	15:77532443-77532675	14.815	0.000	13.428	21.768
Zc3h7a	16:11136694-11176449	18.019	66.577	16.310	16.097
Dusp1	17:26642533-26645639	6.156	10.271	7.292	9.745
AY036118	17:39981973-39985775	78.210	212.687	72.855	73.357
Six3	17:86001750-86025499	0.154	1.102	0.274	0.628
Tcf7l2	19:55816309-56008146	1.157	8.807	1.468	2.675
Htr2c	X:143396975-143631821	6.342	12.437	8.440	9.408
**Fmod**	**1:135933969-135944800**	**1.600**	**3.299**	**3.373**	**3.279**
**Ptgds**	**2:25318620-25321886**	**0.219**	**0.935**	**0.503**	**0.281**
**S100a8**	**3:90473003-90473954**	**7.764**	**2.042**	**1.499**	**1.563**
**S100a9**	**3:90496559-90499221**	**8.422**	**2.628**	**2.099**	**1.431**
**Wdr86**	**5:24216845-24236452**	**0.240**	**3.018**	**0.985**	**2.856**
**BC030500**	**8:61379343-61393163**	**0.909**	**0.000**	**1.982**	**0.228**
**Calml4**	**9:62705887-62723783**	**1.586**	**7.113**	**3.630**	**8.037**
**Narg2**	**9:69245774-69280992**	**0.091**	**0.490**	**0.304**	**0.482**
**Pmch**	**10:87553815-87555214**	**0.373**	**2.791**	**0.155**	**0.105**
**Wfikkn2**	**11:94097301-94104025**	**0.181**	**0.651**	**0.436**	**0.869**
**Dio3**	**12:111517039-111519293**	**0.689**	**1.699**	**2.572**	**1.545**
**4930427A07Rik**	**12:114394889-114403682**	**1.909**	**0.244**	**0.292**	**0.310**
**Gm9307**	**14:103409438-103409867**	**23.347**	**5.840**	**9.150**	**10.242**
**Apol7e**	**15:77532728-77532933**	**66.098**	**8.501**	**5.214**	**7.550**
**Glp1r**	**17:31038889-31077715**	**0.457**	**1.020**	**1.148**	**1.457**

*The values are expressed as Fragments Per Kilobasepair per Million (FPKM) of the gene in sample*.

**Figure 4:**
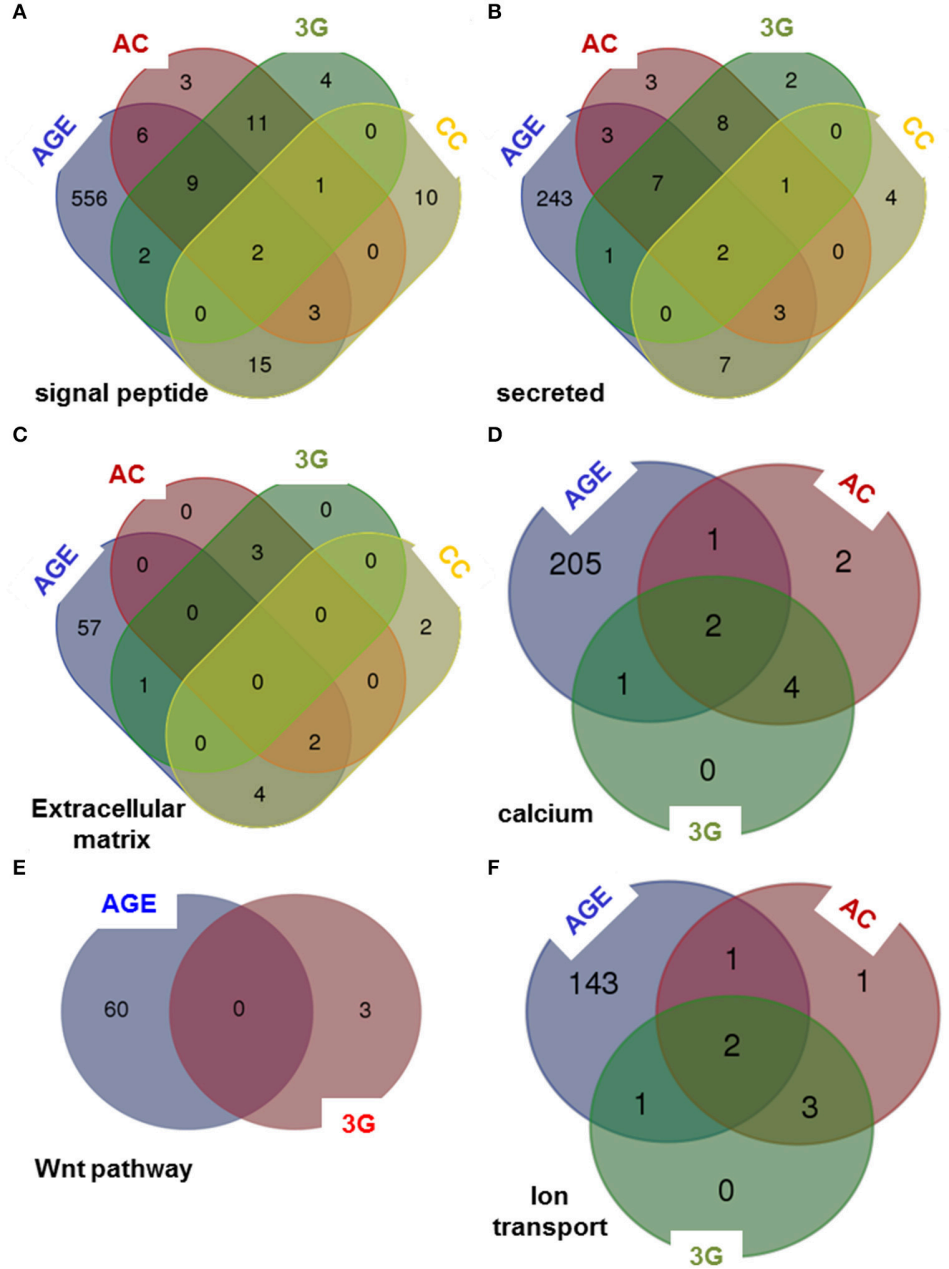
**Venn diagrams comparing the queries of DAVID database**. The lists of genes modified by all experimental conditions were analyzed using DAVID database and Venn diagrams were created considering six categories of genes encoding: **(A)** signal peptide, **(B)** secreted proteins, **(C)** proteins of the extracellular matrix, **(D)** proteins implicated in calcium-dependent mechanisms, **(E)** proteins implicated in Wnt pathway and **(F)** proteins implicated in ion transport.

To compare the putative molecular effects and function of 3G and other experimental conditions, we have crossed the results of DAVID queries to generate Venn diagrams. The effects are not stackable in any functional category proposed by DAVID. As illustrated in Figure 4, some categories are affected by all experimental conditions in Figures 4A–C, whereas some others are modified by only three (Figures 4D,E) or two (Figure 4F) of them. As expected, the aging group is the most affected by the number of gene and the number of function (Supplementary Table 1).

### Verification by RT-qPCR of some gene expression levels

**Figure 5:**
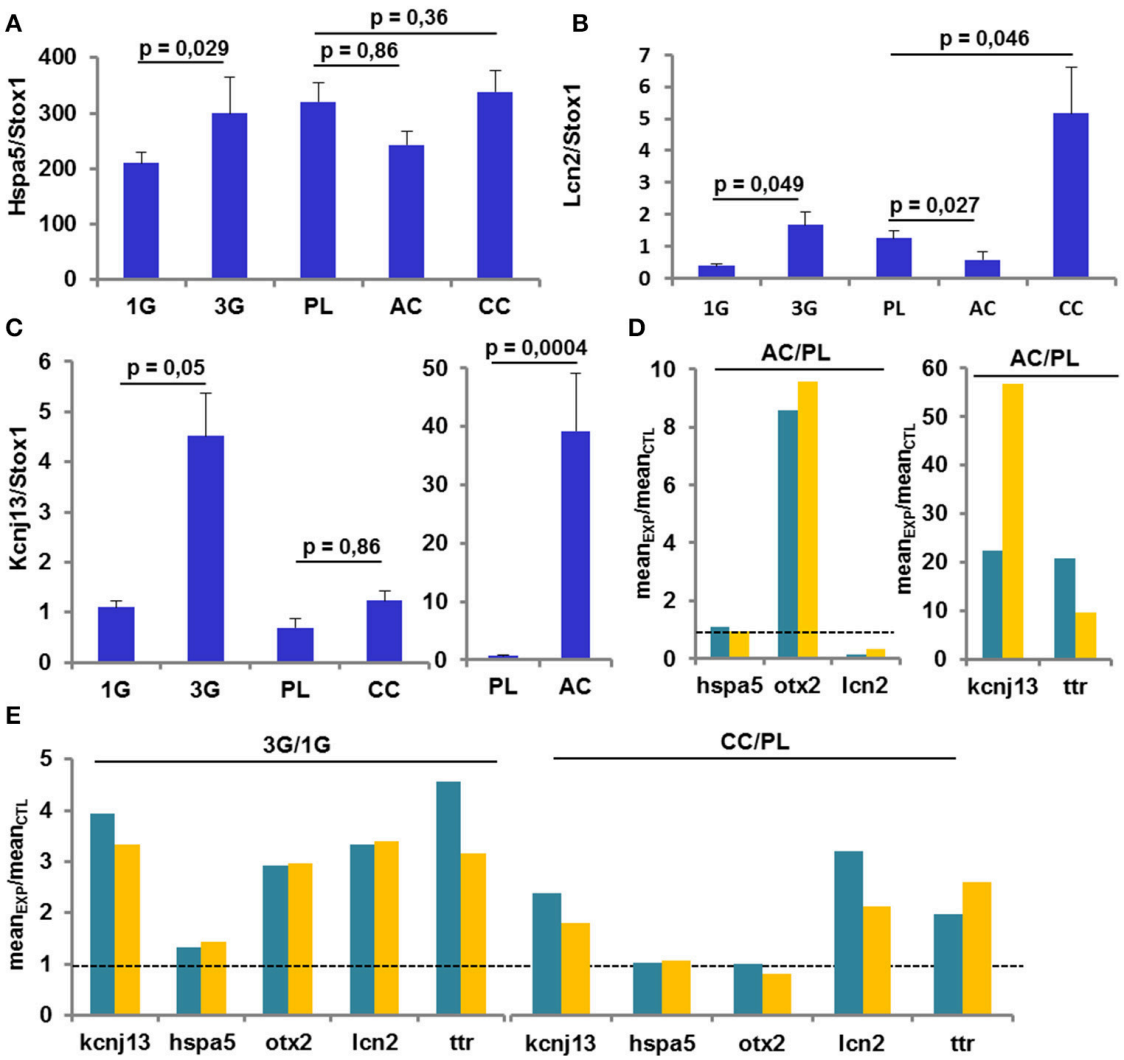
**Gene Expression revealed by RT-qPCR. (A–C)** Mean ± sem of gene expression level quantified after RT-q-PCR in all conditions. The *p*-values indicated were obtained by Student's *t*-test analysis. **(D–E)** comparison of the ratio AC/PL, CC/PL, and 3G/1G obtained with transcriptomic (green) and RT-qPCR (orange) analysis.

The analysis of several gene expression levels was performed by RT-qPCR on the same samples used in the previous set of experiments and compared to the transcriptomic results. As shown by transcriptomic analysis, the expression of Hspa5 was increased only in the 3G condition (Figure 5A); the expression of Lcn2 was increased in 3G and CC conditions whereas was decreased in AC conditions as observed in transcriptomic analysis (Figure 5B). Finally, the Figure 5C, illustrated that the expression of Kcnj13 was increased in AC conditions and was not affected in CC whereas the effect of 3G appeared weakly significant (Figure 5C). The explanation of this last effect could be ascribed to the fact that in 30% of 3G samples the ΔCt values are similar to the ΔCt values measured in 1G group. Anyway, the ratio between each experimental condition and its control revealed that the observed changes point in the same direction (Figures 5D,E) compared to transcriptomic analysis, showing converging evidence.

## Discussion

We confirmed that (1) C57BL6 mice exposed for 21 days to hypergravity showed an increase of their corticosterone plasma levels, (2) the previously reported variability of the individual adaptation to gravity modifications was indeed present (Beraneck et al., 2012; Gnyubkin et al., 2015).

It is interesting to compare our results with those obtained in other species, devices, and protocols. In rats, the level of corticosterone could increase during hypergravity exposure and after the stop of centrifugation (Petrak et al., 2008), but also increased or decreased depending on the hypergravity protocol and age of the animals, as shown respectively in (Casey et al., 2012; Abe et al., 2013). Thus, the design of the hypergravity protocol is crucial. For example, a regular “stop and go” during the centrifugation can lead to a habituation that can reduce the stress effects.

Taken together, all these studies indicate that the design of the centrifugation protocol and the animal model could influence the stress produced by hypergravity. It would be very interesting to test how centrifugation protocols could influence physiological parameters as plasma concentration of several hormones on different species (mouse and rat) to determine general rules in biological adaptation to gravity levels. Finally, these experiments could also reinforce the theory of the gravity continuum proposed and discussed in the biology and gravity fields (Plaut et al., 2003; VanLoon, 2016). Probably, as suggested by these studies, the effect of hypergravity close to 2G is the opposite of microgravity, while the effects induced by 3G acceleration could be different, without following a linear trend. The continuum of gravity should be established independently for each physiological function or tissues.

The absence of handling during the 21 days of centrifugation obliged us to limit the manipulation of mice in the other groups as well; thus the wide range in corticosterone measures and the relatively high corticosterone levels in 1G controls may reflect a release of corticosterone in response to the stress of handling (Pitman et al., 1988). In this work, we decided to consider the PL group as a control group for AC, despite the fact that more adequate controls would have been mice acutely injected with saline; however, the animal sacrifice was performed 1 h after the acute injection, time by which its effects on corticosterone levels were unneglectable (Freund et al., 1988). It is noteworthy that all sacrifices were performed in the same time window, not only to limit the bias due to the circadian rhythm of corticosterone synthesis, but also to decrease misinterpretation linked to circadian rhythm of protein synthesis and gene regulation implicated in memory processes (Eckel-Mahan, 2012).

The chronic treatment with corticosterone increased the level of plasma corticosterone (*p* = 0.042, after the Thompson-test revealing one outlier for 19 mice in both groups), yet in a lesser extent than expected. As reported recently, in order to reach a 1.8- to 2-fold increase of the blood corticosterone concentration in mice after 21 days, a dose of 40 mg/Kg/day would be required (Ali et al., 2015; Weng et al., 2016). In our experiment, the designed pellet delivery was close to 20 mg/Kg/day and the increase of the measured plasma corticosterone concentration was 1.5-fold compared to PL group. Since drug delivery problems can be excluded, this level of blood corticosterone can be due to different turn-over mechanisms.

Interestingly, by comparison with placebo-treated animals, we also observed a slight increase of the corticosterone concentration in group 1G (*p* = 0.052, *t*-test's comparison) close to the ones observed in aging and chronically-treated mice. This result suggests that containment alone can be sufficient to induce a rise in corticosterone levels. Future experiments should clarify if this increase can impact the memory performance by itself (Salehi et al., 2010).

Before our study, several studies reported that aging as chronic stress affect similarly hippocampus function as learning and memory (Tronche et al., 2010; Bonhomme et al., 2014; Wang et al., 2016), then we hypothesized that the modifications of hippocampus transcriptome induced by hypergravity should be similar to those observed in aged mice and/or mice treated with corticosterone.

The hippocampus transcriptome is modified in each experimental condition. Our results show that there is no overlap between experimental conditions but 61, 38, and 5% of transcripts modified by hypergravity were respectively modified by AC, aging, and CC in the same way; whereas 14, 4, and <3% of transcripts modified by hypergravity were respectively modified by aging, AC and CC in the opposite way.

A global analysis of the transcriptome can give a snapshot of the hippocampus status, thereby revealing possible modifications of its functions induced by hypergravity, corticosterone (acute i.p. and long lasting s.c. delivery), and aging. In order to validate our experimental procedure—pooling RNAs from different mice in one sample used in transcriptomic—and to test the biological variability, some gene expressions were analyzed by RT-qPCR.

The data obtained confirmed the effects observed in transcriptomic arrays, suggesting that the experimental design (pooling samples in experimental triplicates) is not able to induce massive bias (Auer and Doerge, 2010; Rajkumar et al., 2015). These data also underlined the effects of hypergravity by increasing the dispersion of individual scores as shown with the corticosterone measurements.

It appears from our result that a very large part of the hypergravity effects on hippocampal transcripts (summarized in Figure 2) is reproducible by an acute injection of corticosterone used to replicate the symptomatology of PTSD (Kaouane et al., 2012). The most probable event comparable to acute stress in our protocol is the stop of the centrifuge. Consequently, we suggest that the stop of the centrifuge is perceived by animals as an acute stress that could induce cognitive disorders close to PTSD. Further experiments should be performed to analyze the memory performance once normogravity is reestablished, using behavioral experiments, such as fear conditioning. Moreover, in order to decrease the effect of rapid gravity modifications as well as to better compare the effects of the gravity in rodents to those seen in humans, animals could be trained to several acceleration training phases or modulation of velocity in both “lift-off” and “landing” phases, etc…Indeed, studies on humans differ largely from the ones using rodents: first, astronauts undergo sustained training before being submitted to space flight; second and most importantly, since humans beings are conscious, they can prepare themselves to the changes of gravity during the mission (i.e., since they know when and why the gravity changes, thus the situation is less stressful).

Surprisingly, the long lasting subcutaneous diffusion of corticosterone used to mimic a chronic stress is the situation that mostly differs from hypergravity, moreover, there only 14 genes affected by both acute and chronic corticosterone treatments. These treatments were used to mimic stress effects and several studies reported the effects of the acute and chronic stresses modified differently the hippocampus transcriptome (Li et al., 2013; Stankiewicz et al., 2015). Furthermore, the nature of the stressor is also crucial (Porter et al., 2012; Li et al., 2013; Suri et al., 2014; Stankiewicz et al., 2015). Similarly to stress, modifications of living conditions, as well as long-term mild or intense exercise, can modulate the hippocampus transcriptome (Inoue et al., 2015). In our case, the effect of the stop of the centrifuge create a higher increase of plasma corticosterone compared to the one observed following chronic corticosterone treatment, and reveals a similar modification of the hippocampus transcriptome to the one observed after an acute injection of corticosterone.

Aging is characterized by a substantial modification of the hippocampus transcriptome (close to 10% of the sequences are affected). This result is probably due to an important dispersion of individual physiological status/history but also to the absence of selection of the mice based on learning and memory performances, for example. Moreover, the number of gene affected by aging depends also on the age of the mice (Stilling et al., 2014). Finally, the lists of genes affected by both aging and corticosterone treatments contain only 55–57 genes, largely different from the hypergravity. These observations underscore that aging as other experimental conditions integrate multiple signals and their complex marks; a transduction pathway can be affected in one or more different steps. Ultimately, it is possible to implicate several identical pathways affected in all conditions as revealed by the results of DAVID queries.

The results obtained in the transcriptome analysis should be evaluated remembering that the hippocampus contains several cell types as neurons, glial cells but also endothelial cells and pericytes constituting blood vessels. A recent study showed that the choroid plexus could be affected by hypergravity and indicated that Ttr, Igf2, Igfbp2, Prlr, Enpp2, Sostdc1, 1500015O10RIK (Ecrg4), Kl, Clic6, Kcne2, F5, Slc4a5, and Aqp1 were more highly expressed in the choroid plexus compared to the brain parenchyma (Stankiewicz et al., 2015). Similarly, in our analysis, we found that the transcription levels of these genes were affected by hypergravity. We cannot totally exclude a bias due to the dissection step. Nevertheless, the effect of gravity modifications on the choroid plexus should be more investigated, because blood and cerebrospinal fluid pressures are both sensitive to hypergravity (Iwasaki et al., 2012).

**Figure 6:**
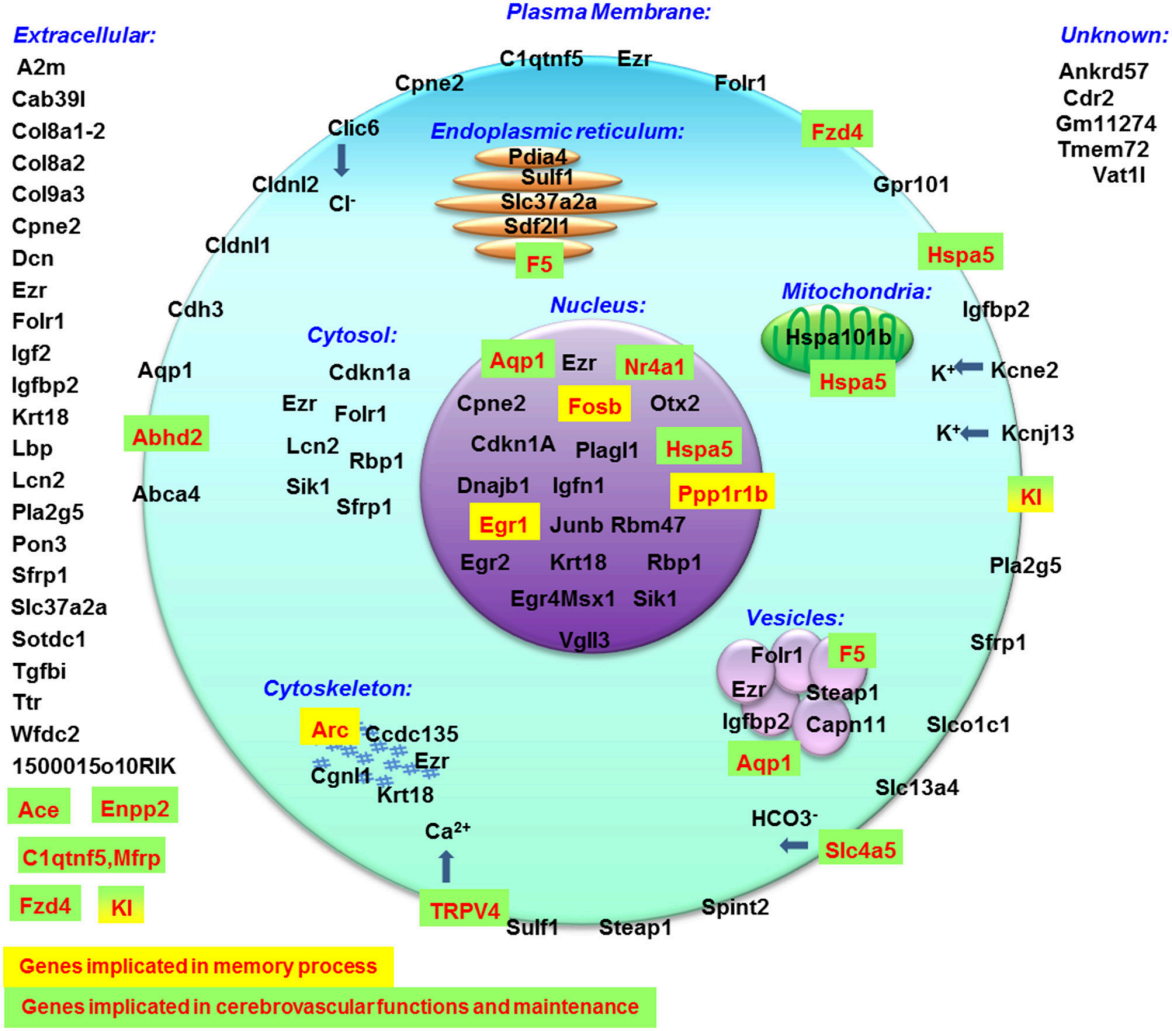
**Cellular localization of proteins encoded by genes modified by hypergravity created with data from MGI database**.

Globally, the query of MGI database indicated that all affected genes are expressed in nervous system and consequently all biological processes could be affected [Supplementary Figure 3; response to stimulus (concerning 46 genes affected by hypergravity), signaling (28 genes), development (30 genes), immunity (15 genes), localization (29 genes), component organization (29 genes), homeostasis (11 genes), cell differentiation (28 genes), proliferation (17 genes) and death (15 genes), protein metabolic process (26 genes), nucleic acid templated transcription (20 genes), lipid metabolism (9 genes), and carbohydrate derivative metabolism (6 genes)]. Furthermore, this database also indicated the cell localization (summarized in Figure 6) and the phenotypic modifications revealed in transgenic mice. Thus this query indicated that memory processes could be modified via the increases of expression of Arc, Fosb, Egr1, Kl, and Ppp1r1b and the blood brain barrier and/or the functions and morphology of cerebrovascular sphere could be affected via the modification of gene expressions of Abhd2, Ace, Aqp1, Enpp2, Fzd4, Kl, Nr4a1, Slc4a5, Trpv4, F5, Hspa5, and C1qtnf5, Mfrp. Surprisingly, only 1500015O10Rik (Ecrg4) is implicated in the control of corticosterone production (Tadross et al., 2010) and produced by choroid plexus. Moreover, the query of DAVID database indicated that genes affected by hypergravity encode for proteins implicated in several functions. Probably, the most important common effects of hypergravity and acute injection of corticosterone concerned signaling pathways affecting nucleus functions and secreted proteins as compounds of extracellular matrix and cellular interactions as modulation of tight junctions. However, the cellular metabolism does not seem to be deeply disturbed, as observed in other studies concerning different organs in a pregnant rat model (Casey et al., 2012, 2015). The effects of hypergravity on hippocampal transcripts are not solely overlapped to acute stress, in fact 10 transcripts were *per-se* modified by hypergravity only. The transcriptomic approach could be used to determine targets potentially affected by environmental modifications (Huttenrauch et al., 2016).

Hypergravity modulate the expression of genes implicated in neuronal differentiation and migration via Plagl1 expression (Adnani et al., 2015). According to DAVID database, the hypergravity can alter learning and memory via the modulation of ppp1r1b, Dnajb1, Rbp1, and Hspa5 genes encoding proteins involved in the regulation of molecular pathways of memory and linked to neurodegenerative disorders (Leil et al., 2003; Ignacak et al., 2009; Heyser et al., 2013; Witt, 2013; Seo et al., 2014; Udan-Johns et al., 2014). The role of CTRP5 encoded by C1qtnf5-Mfrp gene is not described in the brain but it is associated with macular degeneration (Hayward et al., 2003) and lipid oxidation (Yang and Lee, 2014). If the functions of Igfn1 and Krt18 are unknown in neurons, their function in smooth muscle cells (Baker et al., 2010) and implication in intracerebral arteriovenous malformations (Sasahara et al., 2007) indicate that hypergravity could act on pericytes to modify the cerebrovascular function and hippocampus perfusion. Similarly, Sdf2l1, an endoplasmic reticulum stress-inducible gene (Fukuda et al., 2001) is implicated in the folding of proteins (Tiwari et al., 2013) but the function of the encoded proteins in brain cells (neurons, glia, and vessels) remains largely unknown. Finally, sFRP1, encoded by sfrp1 gene, has been found upregulated by hypergravity. It has been shown that the intracerebral injection of this protein antagonized Wnt, resulting in inhibition of bassoon activation (Tabatadze et al., 2014) and the blockade of the CamKIIα phosphorylation, decreasing the fear memory acquisition, and consolidation performances (Xu et al., 2015). Thus this last result can suggest that memory could be altered by hypergravity making essential the study of fear conditioning memory in hypergravity models.

In conclusion, even if it is not completely possible to segregate the effects of stress and hypergravity in this model, the transcriptomic analysis indicated that the expression of genes encoding proteins implicated in excitability, cellular interactions, migration, protein synthesis, and cell death, was modified after 3G exposure. An effect on memory thus cannot be excluded since memory requires a coordinated regulation of many known and unknown proteins (Jarome and Helmstetter, 2014). It is also known that stress affects deeply the cerebrovascular network (Scheuer et al., 2007; Longden et al., 2014). Therefore, a modulation of the vascular network, due to hypergravity, should not be totally excluded and could act on brain functions especially in animal models without training as in astronauts (cognitively, emotionally and physically). Considering that 10 genes were modified specifically after 3G exposure, it appears that hypergravity alone modulates the hippocampal activity. We can thus suggest that both the neurogenesis and angiogenesis would be impaired (decrease of plagl1), whereas neuroprotection against hypoxia effects would be ameliorated (increase of rbp1 and dnajb1). Moreover, the increases of expression of factors regulating protein folding (sdf2l1, hspa5) and NMDA-dependent signals (ppp1r1b) most probably affect the neuronal architecture. Finally, our results could also be compared to other studies using the same approach to understand how the hippocampus transcriptome was adapted to environmental conditions (Huttenrauch et al., 2016).

## Author contributions

The project was initiated by YP continued by AP under supervision of JM. YP and JM prepared samples; AP and JM analyzed the data and JM wrote the manuscript. All authors approved the manuscript.

## Funding

The study was supported by grants from Centre National des Etudes Spatiales (CNES; grants n° 007088, 007636) and Agence Nationale pour la Recherche (AdapHyG n° ANR-09-BLAN-0148). YP was granted by ANR and AP's doctoral fellowship was granted by CNES and Region Aquitaine.

## Conflict of interest statement

The authors declare that the research was conducted in the absence of any commercial or financial relationships that could be construed as a potential conflict of interest.
