# Supplementary figures and images for: Changes in C57BL6 Mouse Hippocampal Transcriptome Induced by Hypergravity Mimic Acute Corticosterone-Induced Stress
Source: Front Mol Neurosci. 2016 Dec 26;9:153. doi: 10.3389/fnmol.2016.00153 (PMC5183579; doi:10.3389/fnmol.2016.00153)

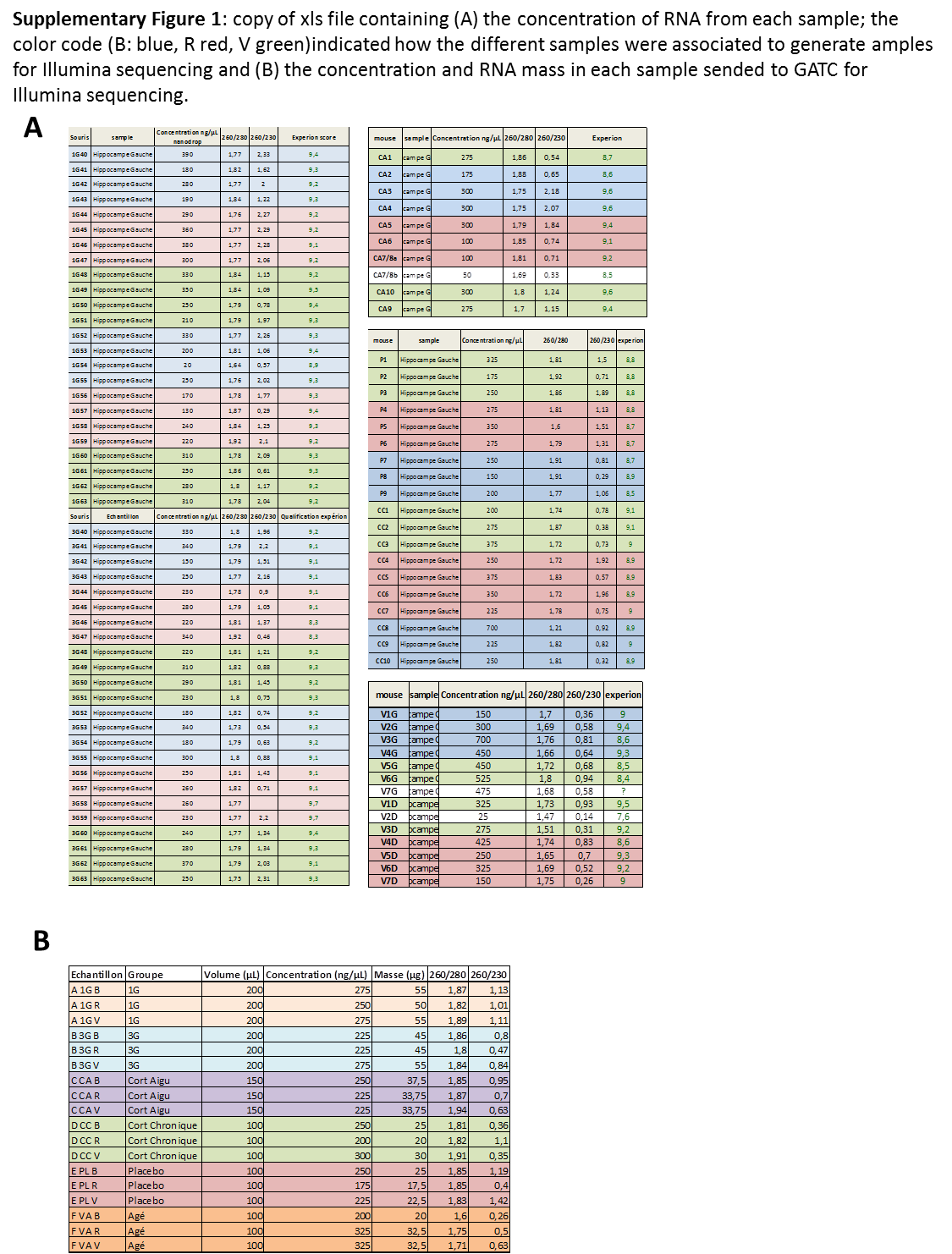

Supplement: Supplementary file 3 [file Image1.TIF]

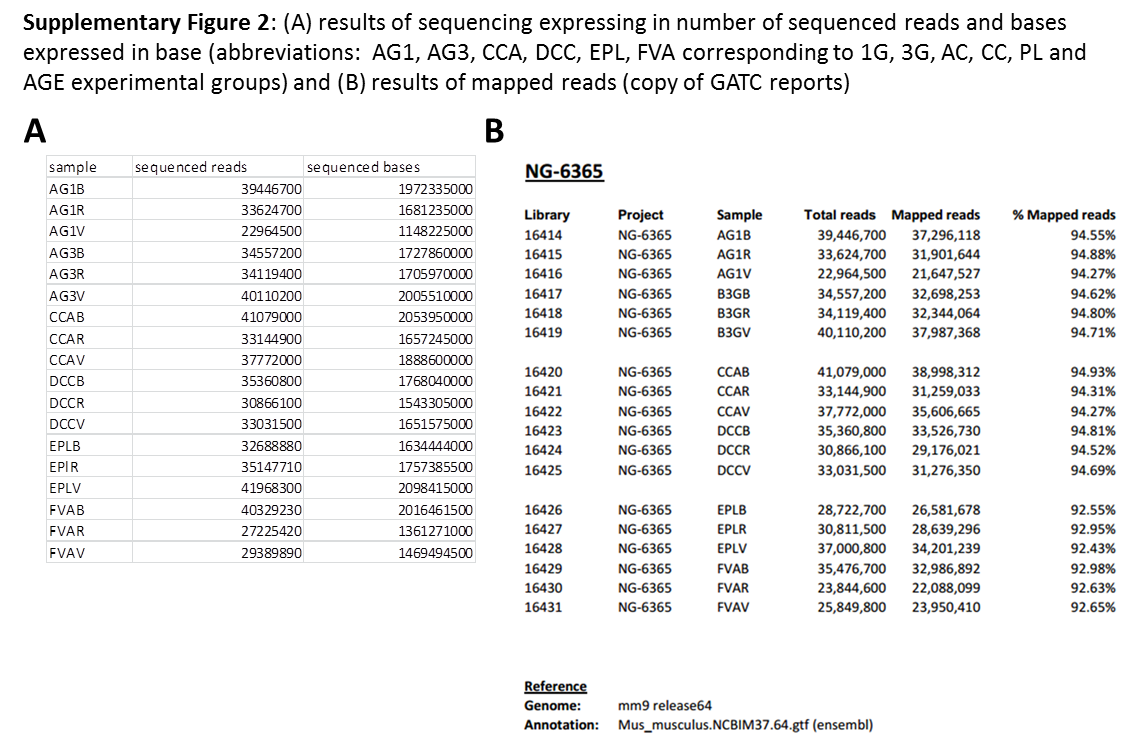

Supplement: Supplementary file 4 [file Image2.TIF]

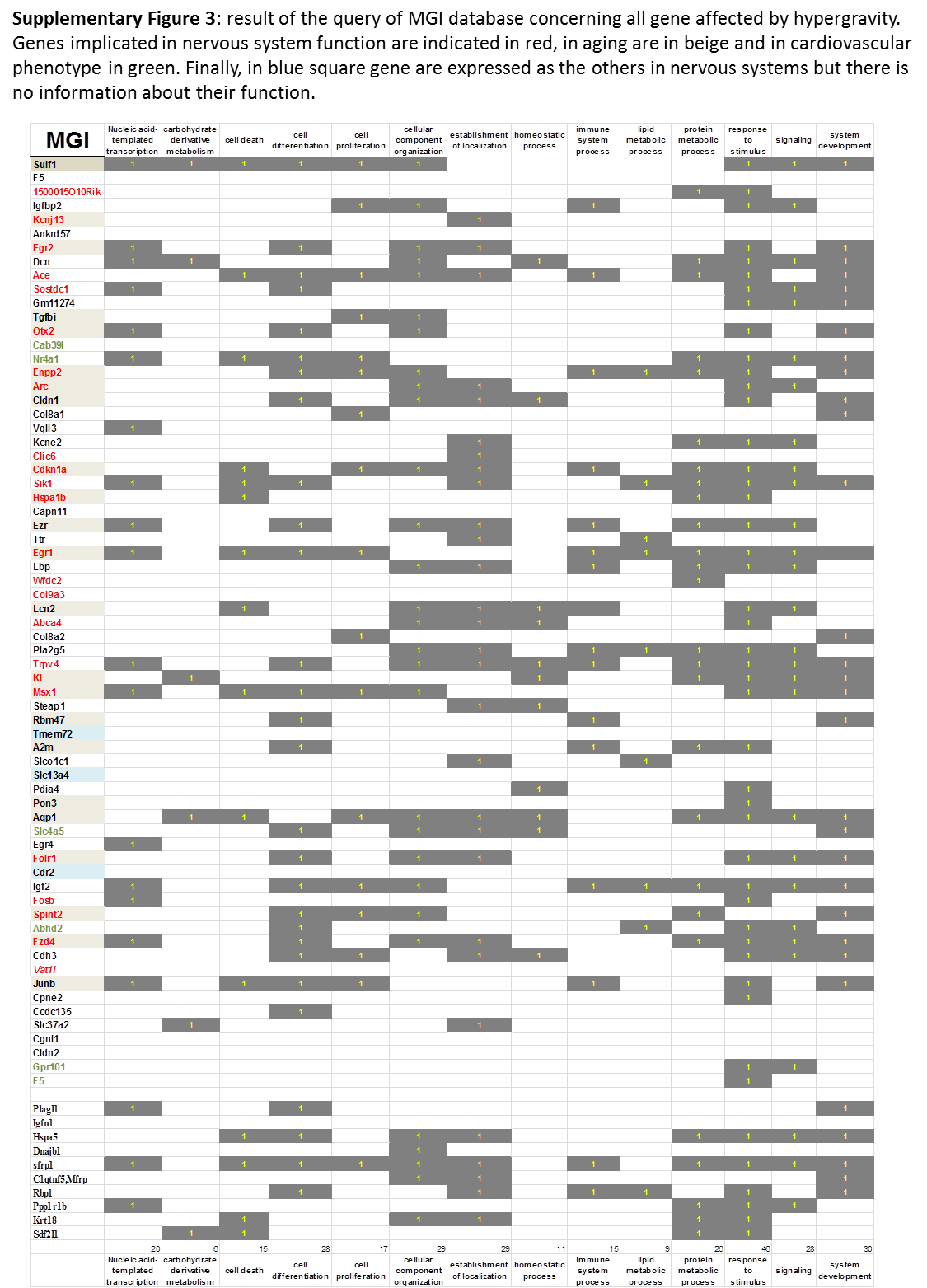

Supplement: Supplementary file 5 [file Image3.TIF]
